# Penile implant infection resulting in *Staphylococcus aureus* bacteraemia and infective endocarditis

**DOI:** 10.1099/acmi.0.000295

**Published:** 2021-12-09

**Authors:** Joseph P. Creel, David Triplett, Mannu Nayyar, Nathan A. Summers

**Affiliations:** ^1^​ Department of Medicine, University of Tennessee Health Science Center, Memphis, TN, USA; ^2^​ Division of Cardiology, University of Tennessee Health Science Center, Memphis, TN, USA; ^3^​ Division of Infectious Diseases, University of Tennessee Health Science Center, Memphis, TN, USA

**Keywords:** bacteraemia, endocarditis, penile implant, echocardiogram

## Abstract

**Introduction:**

Penile implant infections are a possible surgical complication that has historically been most commonly associated with Gram-positive bacteria. *Staphylococcus aureus* is a Gram-positive bacteria and is the most common cause of endocarditis.

**Case Presentation:**

A male patient in his 50s with a past medical history of hypertension, diabetes, end-stage renal disease (ESRD) on peritoneal dialysis (PD) and erectile dysfunction with a penile implant placed 6 years prior to the admission date presented with complaints of scrotal pain. The pump for his implant had eroded through his scrotum and was draining pus. Blood cultures returned positive for Gram-positive cocci in clusters in 4/4 bottles, which was eventually identified as methicillin-sensitive *Staphylococcus aureus* (MSSA). A transthoracic echocardiogram (TTE) was performed due to concern for infective endocarditis (IE) but did not show any valvular abnormalities. Due to high clinical suspicion, a transesophageal echocardiogram (TEE) was performed and revealed a vegetation on the native mitral valve. His penile implant was removed by urology and intraoperative cultures grew MSSA. Surgical valve replacement was not recommended, and the patient was sent home with IV antibiotics for 6 weeks.

**Discussion:**

Post-operative site infections are a quite uncommon point of entry for infective endocarditis, with penile implant infections being an even rarer cause. While a TTE is often used initially to attempt to diagnose infective endocarditis, it has lower sensitivity than a TEE. If clinical suspicion for infective endocarditis remains high after negative imaging with TTE, then TEE should be performed for better visualization of the heart valves.

## Introduction

Infection of implanted hardware is a rare but unfortunate complication of penile implants that occurs in 1–3% of cases [1]. Historically, the organisms most commonly associated with penile implant infections were Gram-positive bacteria, more specifically *Staphylococcus* species. In 2001 penile implants that contained infection-retardant coatings were introduced [2]. While this has caused an increase in the diversity of the organisms in these cases, with a notable increase in infections caused by fungi and Gram-negative bacteria, Gram-positive organisms are still most often identified as the cause [1].

Infective endocarditis is the infection of a heart valve, the endocardial surface, or an implanted cardiac device. Echocardiography is key to diagnosis of this disease [3]. Transthoracic echocardiography (TTE) is often obtained first when there is clinical suspicion of infective endocarditis because it is more easily available to most patients while also maintaining moderate sensitivity (70%) [4] and good specificity (>90%) [3]. Transesophageal echocardiography (TEE) has greater sensitivity (>90%) and is required to make the diagnosis of infective endocarditis in some cases, such as non-diagnostic TTE images, the presence of prosthetic valves or an intracardiac device, or high clinical suspicion for infective endocarditis following a negative TTE [3].

Gram-positive bacteria are also the most common cause of endocarditis, with *Staphylococcus aureus* causing 40% of valve infection in the US in 2011 [5]. Understanding the possible connection of these two different types of infection will be explored in this case study.

## Case report

Our patient was a male in his 50s with a past medical history of hypertension, diabetes, end-stage renal disease (ESRD) on peritoneal dialysis (PD) and erectile dysfunction with an inflatable penile prosthesis (IPP) placed 6 years prior to the admission date who presented with complaints of scrotal pain.

The pump for his IPP had eroded through his scrotum and had been intermittently draining pus for the 2 months before presentation. He also noted fevers, chills, nausea and vomiting over this same time period. He stated that he had been treated at an outside hospital 2 weeks prior to this admission but was only given a prescription for pain medication and discharged home without antibiotics. Upon arrival at the emergency department, the patient was febrile to 39.0 °C and was tachycardic with a heart rate of 101. Infection was suspected, and the patient was started on vancomycin and cefepime. Blood cultures were obtained, and a CT of his abdomen and pelvis was done that showed a soft tissue scrotal defect and surrounding inflammatory changes without evidence of abscess. Urology was consulted and planned for explant of the prosthesis. The patient was also admitted to the internal medicine service as the primary team and moved to the hospital wards floor.

**Fig. 1.**
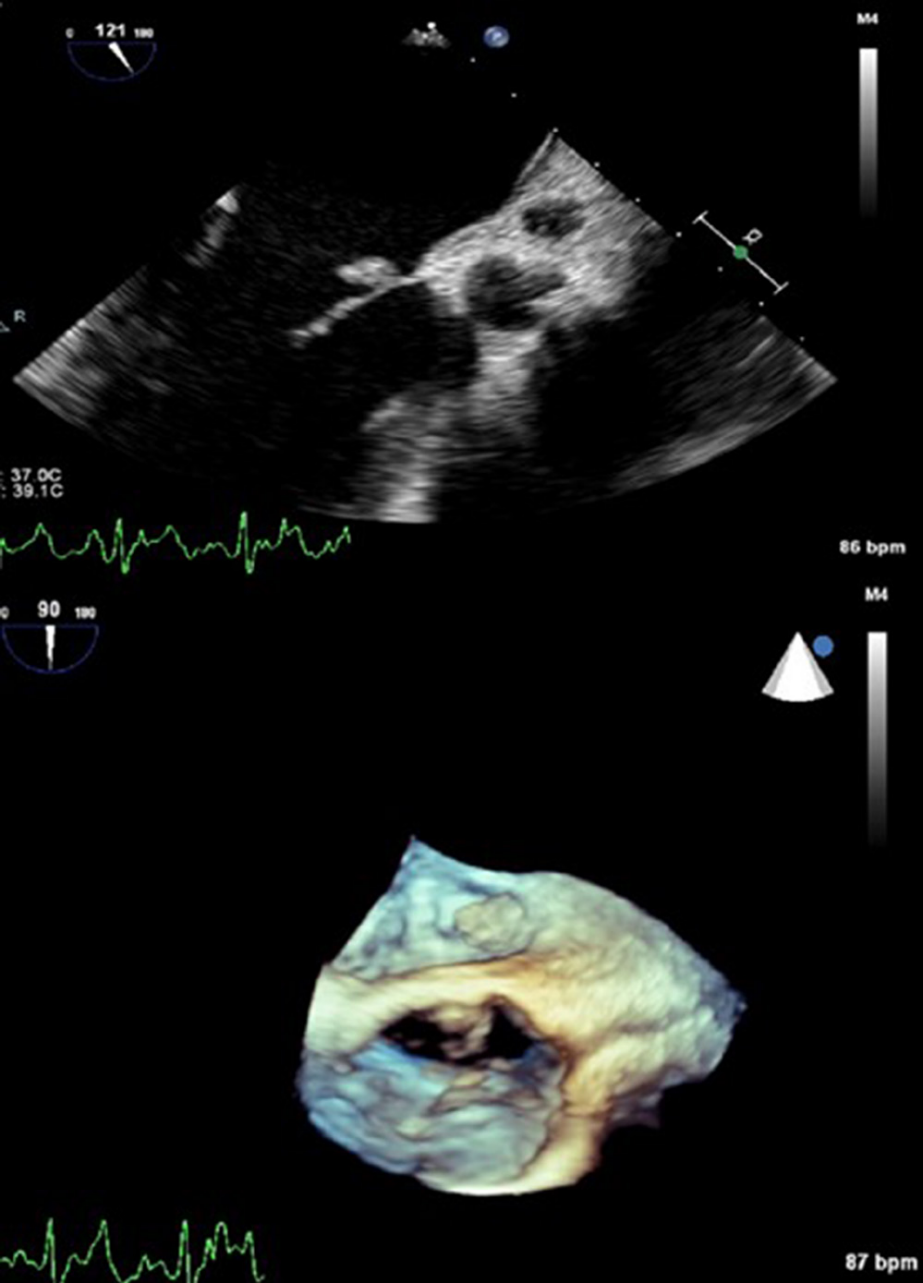
Transesophageal echocardiogram images of the mitral valve revealing a 1.1×0.7 cm mobile vegetation.

Blood cultures returned positive in 4/4 bottles for Gram-positive cocci in clusters. Infectious Disease was consulted and recommended transthoracic echocardiogram (TTE) and repeat cultures. TTE was done and showed no valvular abnormalities, notable only for mild mitral regurgitation. In light of the prolonged duration of symptom of fever for 2 months before presentation and concern that he may have been bacteraemic for an extended time prior to admission, there was a high level of concern for infectious endocarditis. A transesophageal echocardiogram (TEE) was done and revealed a 1.1×0.7 cm mobile lesion located on the atrial side of the anterior mitral valve leaflet (Fig. 1). Cardiology and Cardiothoracic surgery were consulted and did not recommend valve replacement surgery.

The patient’s fever and tachycardia improved the day following administration of antibiotics, and he remained afebrile for the remainder of his hospital stay. Blood cultures and intraoperative cultures from the explanted inflatable penile pump (IPP) returned positive for methicillin-sensitive *S. aureus* (MSSA). Antibiotics were changed to cefazolin. His blood cultures cleared shortly after removal of the IPP, and the patient was discharged home on cefazolin to complete a 6-week course. The patient followed up in the outpatient clinic 2 weeks following discharge and reported improvement of his symptoms. Unfortunately, he passed away from an unrelated subdural haematoma 3 weeks later. There was no evidence of sepsis during this subsequent hospitalization.

## Discussion

Penile implant infections are an uncommon but serious potential complication following surgery. Studies have shown that most penile implant infections occur within the first year after surgery [6]. However, this case demonstrates that there is still possibility for infection in the years following surgery.

When infective endocarditis (IE) is diagnosed, it is important to identify a potential origin of the infection. The majority of cases of endocarditis are caused by community-acquired infections, with only around 30% being caused by healthcare-associated sources. Within this smaller subset, the origin of the infection is usually a vascular access site. Only ~11% are associated with post-operative sites, with the most common causes being cardiac valve replacements, vascular surgeries and orthopaedic surgeries [7]. The patient described in this case report had a very uncommon site as the point of entry for his infective endocarditis.

Imaging with echocardiography is important when attempting to diagnose infective endocarditis [8]. TTE is often used first due to being more easily accessible and does not require the patient to undergo a procedure with general anaesthetic, as is the case with TEE. However, TTE only has a sensitivity of 70% when evaluating native valves and 50% when evaluating prosthetic valves. TTE also has more difficulty in visualizing vegetations on valves with pre-existing lesions [4]. TEE has a higher sensitivity of 94% for diagnosing infective endocarditis [9]. While TTE is often used first, it is important to understand that the lower sensitivity of this test can cause a potential case of IE to go undiagnosed. If initial imaging with TTE is negative and clinical suspicion for endocarditis is high, as with the patient in the case, then a TEE should be performed afterwards to better visualize the valves [4].

One of the factors that led to obtaining a TEE in this case was the fact that the patient was found to have MSSA bacteraemia upon presentation. Community-acquired bacteraemia has previously been shown to be associated with an increased risk for IE [10, 11]. One reason for this association is related to the higher risk of IE when the duration of bacteraemia is prolonged (typically considered >72 h) [11]. Because our patient had symptoms for 2 months prior to his presentation, there was significant concern that his bacteraemia, which was found on admission, may have been present for a protracted course. As such, TEE was pursued despite the relatively normal findings on TTE and did ultimately reveal a mitral valve vegetation.

As seen in this case, infective endocarditis is an interesting disease that can sometimes originate from an uncommon source. Diagnosis can be complicated by inability to visualize vegetations on TTE, so it is important to understand the necessity of obtaining a TEE if clinical suspicion is high, particularly with community-acquired bacteraemia.
